# Pros and cons of different stunning methods from a Halal perspective: a review

**DOI:** 10.1093/tas/txab154

**Published:** 2021-10-11

**Authors:** Mian N Riaz, Fariha Irshad, Nooran M Riaz, Joe M Regenstein

**Affiliations:** 1 Department of Food Science and Technology, Texas A&M University, College Station, TX 77843, USA; 2 Department of Food Science, College of Agriculture and Life Science, Cornell University, Ithaca, NY 14853-7201, USA

**Keywords:** animal welfare, halal, slaughtering, stunning

## Abstract

There has always been a debate about the acceptability of stunning methods for preparing Halal slaughtered meat. Throughout the last few decades, stunning methods have become acceptable for Halal slaughtering due to an increasing majority of Muslim countries issuing Fatwas (religious rulings) that approve of stunning methods for the Halal slaughtering of food animals. With an increasing Muslim population worldwide, Halal meat provision is important for Muslims both economically and ethically. Moreover, there have been concerns regarding traditional Halal slaughter without the use of stunning from the standpoint of the animal’s welfare. This article reviews the different stunning methods available and the associated processing practices, addressing their pros and cons in the commercial production of Halal meat.

## WORLDWIDE HALAL MEAT MARKET

It is estimated that the Muslim population ranges between 1,600 and 1,800 million people and is growing. It is projected that by 2030 it will constitute 27% of the world’s population (Sungkar, 2008; Farouk, 2012). Islam is growing at a fast pace as the Muslim population in 2017 was estimated to be 1.8 billion, which makes up 24.1% of the world’s population (Hackett et al., 2017). In the United States, the Muslim population constitutes of about 8 to 10 million people and 16 to 20 million Halal consumers (Thomson et al., 2019).

According to the global Islamic economy report for 2019/2020 by Thomson and Dinar, Muslims spent $2.2 trillion U.S. dollars on Halal food, pharmaceuticals, and other sectors in 2018 showing a 5.2% year-on-year growth that is estimated to reach $3.2 trillion U.S. dollars in 2024 at a 6.2% cumulative annual growth rate. Almost 16.6% of all global expenditures for foods and drinks is spent by Muslims, it was estimated to be $1.6 trillion in 2018 and is forecast to grow by 6.3% per year to reach $2.0 trillion by 2024. Disposable income and growing economic development have increased Muslim expenditures. These 2 factors are the main driving forces contributing toward the growth of the Halal market, and potential reasons behind Halal becoming the world’s largest identifiable marketing category. The opportunities it represents, the world’s food giants, and the small and medium enterprises involved in the manufacture and marketing of their products for Muslims around the world makes it a dominating upcoming global market (Nakyinsige et al., 2013).

One of the most important categories and one that is integral to Halal religious practice is the slaughter of animals for food. Traditional slaughter has been done for centuries without “stunning”, i.e., the use of a system to render the animal unconscious prior to undertaking the Halal slaughter of animals. When used for Halal slaughtering, stunning must be reversible, i.e., if not slaughtered, the animal needs to able return to full consciousness and must be able to return to its normal life. Many Muslim countries have acknowledged stunning as a Halal verified technique, which has led to a large number of animals being stunned for the Halal meat market. So much so that it is now estimated that 84% of poultry, 75% of cattle, and 63% of sheep and goats are being stunned as part of Halal slaughtering (FSA, 2015).

## STUNNING

The stunning of an animal before slaughtering is widely practiced around the world. Stunning can be defined as a technical method of making animal immobile or unconscious, with or without killing them, during or at the beginning of the slaughtering process in a way that slaughtering thereafter causes no fear or pain to the animal (Nakyinsige et al., 2013). Another objective is to reduce mobility for easier and safer manipulation by humans of a large animal to aid in the proper and efficient exsanguination. In recent years, from the animal welfare perspective, stunning has been primarily viewed as a mean of minimizing the fear, pain, and discomfort experienced during the time of slaughtering by the animal as long as the stunning is done correctly (Bergeaud-Blackler, 2007).

This is exactly what Islam advocates and acknowledges about the intrinsic worth of humane treatment, especially before and at the time of animal slaughtering. Prophet Muhammad (peace be upon him) stated that: “Verily Allah has prescribed Ihsan (perfection and kindness) in all things. So, if you kill then kill well, and if you slaughter, then slaughter well. Let each one of you sharpen his blade and let him spare suffering to the animal he slaughters” (Sahih Muslim, 40 Hadith Nawawi 17).

Stunning is a relatively new method which was first used a few centuries after the Quran was revealed. Therefore, it would not be expected to be referenced in the sacred writings. In a circumstance where an innovation is not found in the sacred texts, Islamic legal advisers must authorize a judgment (Fatwa) to acknowledge whether that innovation is Halal or not (Fuseini et al., 2016). Several verses in the Quran and numerous narrations by Prophet Muhammad (peace be upon him) emphasized the humane treatment of animal so stunning can be objectively argued as Halal if it induces unconsciousness without causing death (Nakyinsige et al., 2013).

The need to stun, i.e., to induce unconsciousness, before slaughtering relies basically upon 2 principles: (1) animals are sensible creatures; (2) pain and fear for the animal during slaughtering can be avoided by stunning beforehand, if the stunning itself is not painful. Therefore, most animal welfare regulations and guidelines associated with the slaughtering of animal embody an inventory of identified stunning techniques that are appropriate for different animal species and each method specifies a minimum standard to induce immediate unconsciousness that can last until the death of the animal that happens with bleed-out (Bergeaud-Blackler, 2007).

To achieve this in livestock, many Islamic authorities approved different stunning techniques such as a physical blow or passage of an electrical current through the head directly or through an electrified water bath or to induce unconsciousness gradually with a hypoxic or anesthetic gas with different farmed mammals and birds to render them unconscious at the end of the procedure, meaning relatively immediately (Orban et al., 2015; Fuseini et al., 2018). Practically, stunning methods can be faulty and done poorly if equipment is deficient or workers are not properly trained and managed (Anil et al., 1998), and preslaughter handling could expose animals to bad welfare (Warriss, 1998; Warriss et al.,1998). An inhumane slaughter can occur when the animal is still conscious and sensible, or its brain is still functioning fully or even partially to perceive fear or pain. Therefore, the suitable stunning methods for the different types of animals should be decide upon carefully with attention to many details including the following aspects: (1) the desired quality of the final product (meat quality); (2) aspects of animal welfare; and (3) religious requirements, i.e., Halal or Kosher (Bergeaud-Blackler, 2007).

Livestock stunning before slaughtering is done with the goal of ensuring that the animal does not feel pain during slaughtering. There are defined principles based on research underlying effective stunning conditions for livestock which are often regulated by the state. Many such regulations acknowledge the need for good animal welfare to protect these animals and some regulations recognize the special needs for the religious slaughter of animals. Although these legislative and regulatory approaches will vary from country to country, they are largely a reflection of the historical adaptations of local trade methods with minor differences (Farouk, 2013). Development of noninvasive pain reduction methods continue to develop. It remains to be seen if some of them will find a use in the Halal meat slaughtering process in the future (Nakyinsige et al., 2013).

The purpose of this review is to critically look at the different stunning methods with respect to animal welfare and final product quality. The suitable stunning methods with respect to different grades and types of the various animals and birds used for food are reported. Furthermore, this review will try to determine critically the information about whether the slaughter process without stunning is suitable or not, and will also try to identify knowledge gaps and any areas that are not supported by previous scientific research to enable recommendations for the future improvement of animal welfare.

## PRESLAUGHTER STUNNING

Most Halal animals are slaughtered by cutting the major blood vessels, i.e., carotid arteries and jugular veins along with the esophagus and trachea at the neck below the larynx to ensure rapid and complete blood loss. Animals become unconscious after a certain amount of blood loss and with time as blood loss continues, the animal dies, i.e., becomes insensible which is taken as being equivalent to brain death. Animals can experience fear, stress, and pain during cutting of the major blood vessels until unconsciousness (Gregory, 2007). According to the brain and behavioral responses, loss of consciousness occurs in 14 s in sheep by severing both jugular vein and carotid arteries without stunning, whereas it took 70 s by severing only 1 jugular vein and 1 carotid artery. When jugular vein was cut separately, it took 5 min approximately to loss the evoked responsiveness (Gregory and Wotton, 1984a; Gregory et al., 2010; Gibson et al., 2015). According to Newhook and Blackmore (1982), the onset of insensibility was delayed for 29 s when sheep was slaughtered by cutting carotid artery and jugular vein on one side of neck as observed through Electroencephalograms. Stun to neck cut time in goats should be <20 s to avoid the goats regaining consciousness before bleeding. The neck cut should be made immediately after stunning and it is estimated that the acceptable time limit be within 12 s for calves and 23 s for cattle (Grandin, 2020b). Stunning is required to minimize any pain associated with the slaughter of animals and the selection criteria for best stunning methods depends on the animal species (Grandin, 2010). Numerous preslaughter stunning techniques are used in the meat industry for animal slaughtering.

### Electrical Stunning

Electrical stunning is one of the most widely used stunning method as it is economical, convenient for high yields, can be automated and humane for appropriate animals (Gregory, 2007). The primary objective of electrical stunning is neuron depolarization through the passage of an electric current to the brain to subsequently create an uncoordinated movement to make animal unconscious to pain (Sabow et al., 2017). Stunning can be reversible or irreversible by inducing heart failure depending on the time and frequency of the current passing through the brain (Grandin, 2010). Practically, head-only stunning can be reversible while head to back stunning is usually not reversible because of onset of loss of epileptiform activity and induction of anoxia in the brain causing epileptic seizure that eventually produces cardiac fibrillation and induces cardiac arrest (Gregory and Wotton, 1984b).

Electrical stunning causes unconsciousness by generating an epileptic seizure inside the brain. The epileptic seizure condition is comprised of 3 phases: (1) phase 1—fully developed epileptic seizure, (2) phase 2— suppressed brain activity, and (c) phase 3— recovery phase (Farouk, 2013). Meanwhile, normal activity of the brain slowly and gradually recovers and consciousness returns, unless this is stopped by exsanguination.

For stunning to be done effectively, the electrodes must be placed correctly to allow the flow of current to the brain. For effective electrical stunning the amperage will vary according to each animal species and the ideal value is estimated to be 1 amp for sheep and 1.5 amp for cattle (Grandin, 2020b).

### Parameters for Electrical Stunning

Various electrical stunning parameters are utilized throughout the world to accomplish successful stunning of the animal. The electrical stunning method’s viability can be established using 1 of 2 different approaches: (1) by observing the physical behavior of the stunned animal until the time of slaughter or (2) by examining the efficiency of the stunning equipment used for the electrical stun (Gilbert et al., 1984; Lambooy, 1982). In a research study by Gregory (2007), an important requirement for stunning is the inspection of the equipment to ensure that the recommended amount of current with proper positioning of the electrodes has been transferred to the species and then to examine the animal’s behavior after stunning. A typical strategy for evaluating the development of insensibility of a stunned animal is the loss of a corneal reflex, which shows the responsiveness of the brainstem after stunning (Grandin, 1980).

Animals that are irreversibly stunned properly should show the signs of insensibility or no brain activity while passing along the bleeding rail. Reflexes such as periodic breathing, eye movement in response to touch and light, eye flickering, arched reflex with head tilted back, and vocalizations are major indications of improper irreversible stunning. The proper practice of electrical stunning of sheep and cattle can be evaluated by grading at least 100 animals in bigger plants and 50 in smaller ones or 1 h of production in very small plants and determining the following: (1) excellent stunning is achieved when 99.5% to 100% correct placement of the wand or stunner clamps are properly placed with no vocalization because of the electrode activation before it is solidly situated, (2) acceptable stunning is when 99% to 99.4% meet the above criteria and <1% of animals vocalize in response to the placement of the electrodes, (3) stunning is not acceptable when 96% to 98% meet the above criteria and 2% to 3% of the animals vocalize, and (4) serious issues need to be addressed when <96% meet the criteria or >4% of the animals vocalize (Grandin, 2005).

#### Halal requirement for electrical stunning.

Two most commonly used electrical stunning methods are: head-to-body including head-to-back, head-to-forelegs or split current electric stunning, and head-only stunning. Each has different impacts on the stunned animal. When head-to-body stunning is applied correctly, it stops the heart of the animal and causes death, whereas head-only stunning makes the animal unconscious so it does not feel pain and the animal can completely recover come back to its normal state if the exsanguinations cut is not done. Quickly following a head-only stunning, no autonomous movements or responses can be observed as a result of any harmful stimulus. Within 20 to 40 min, animals can return to their normal conscious state showing no symptoms of pain or aversion to stunning (Cook et al., 1995; Velarde et al., 2003). For that reason, head-only electric stunning is considered Halal by many Muslims and is accepted as humane for animals, and virtuous and safe for workers as well (Farouk, 2013).

Head-only electrical stunning when done right is painless from the initiation stage throughout stunning and it does not kill the animal. The physiological response to head-only stunning for sheep was studied by a number of researchers to decide if it is painful to the sheep and they came to the conclusion that the initiation phase of electrical stunning was painless (Leach et al.,1980). A strong synergistic effect is obtained when head-only electrical stunning is followed by the Halal throat cut, i.e., a quick unconsciousness followed with irreversible death. Therefore, head-only electrical stunning is a suitable technique to fulfill the Halal industrial meat processing requirements (Vogel et al., 2011). An important requirement is that the animal still be alive at the time of the Halal slaughter cut. In research studies, this is possible to control, but it is not as easy to assure that this is the case in an actual slaughterhouse where the animals are of different sizes and composition (e.g., lean/fat ratios) along with different amounts of outer coverings, e.g., hair/wool, that varies in thickness and dryness. It is strongly recommended to slaughter the animal (sheep, cattle, and calves) within 15 s after the head-only stunner is removed (Anil, 2012; Grandin, 2020b).

The Gulf Standard Update (GSO 993/1998) contains the basic requirements for the animal’s Halal slaughtering for GCC members. Malaysia, Indonesia, and the Islamic Food & Nutrition Council of America specifies those Halal slaughter standards that include: (1) at the time of slaughtering the animal must be alive; preslaughtering management, such as prestunning, should not cause the animal to die before slaughter. Stunning should be reversible, i.e., the animal must be able to recover completely if not slaughtered; (2) the slaughterer must pronounce the name and glorification of Allah (God) at the time of slaughtering of each animal; (3) a very sharp knife should be used to slaughter animal with one continuous stroke; (4) slaughtering should be done from the anterior to the neckand not from the posterior toward the back; (5) the head ought not be cut off from the neck during slaughtering of the animal so that it may bleed out completely and; (6) carcass handling such as skinning or cutting hocks, is not permitted to begin until the animal is completely dead (insensible), which as a practical matter is the loss of the eye reflex (Riaz et al., 2004).

However, there is still some concern about the humaneness of electrical stunning. Modern experiments are in practice to observe human response to a stimulus and human models can be used to see the response to electrical stunning. Human electroconvulsive therapy (ECT) which is used to treat intractable depression in humans is comparable to electrical stunning in animals but ECT is never been used in its “unmodified” form because of its negative aspects and medical science considered it a torture. This raises questions regarding the use of electrical stunning in the slaughter of animals (Zivotofsky and Strous, 2012).

#### Postslaughter stunning processes related to the production of Halal red meat.

New Zealand follows the following outlines for Halal slaughtering using head-only electrical stunning: (1) a head bail containing the electrode attached to the head of the animal behind the ears and nose tip; (2) an electric current of 1.5 to 2.5 A, 400 V alternating current has been applied across the animal’s brain for 2 to 4 s; (3) the animal falls onto a crib or a mobile table and the Halal cut is executed as soon as possible, but generally within 10 to 15 s after stunning; (4) electro-immobilization electrodes are attached to the animal between the nose and the anus and is switched on passing a 80 to 90 V direct current, 10 pulses at 15 pulses/s; (5) after 15 to 30 s the electro-immobilization electrodes are turned off; (6) the weasand or esophagus is found, clipped, and the animal is shackled so that immobilization is stopped; (7) the trachea/windpipe is rodded and the animal is stuck thoracically and; (8) normal dressing begins (Gilbert et al., 1984). Electro-immobilization and thoracic stick are debatable procedures from the Halal and animal welfare viewpoints (Farouk, 2013) Electro-immobilization has been disapproved because of its potential to mask inappropriate preslaughter stunning (EFSA, 2004) and the thoracic stick was the subject strong concern by some importing countries known for their competent Halal authorities because it is not equivalent to a horizontal neck cut (Farouk, 2013).

#### Positive electrical stunning outcomes.

Electrical stunning is convenient and the most commonly used stunning method as it requires less equipment installation (Grandin, 2020b). High-frequency head-to-body is an advanced form of electrical stunning that has been designed to overcome the flaws in the traditional electrical stunning methods. It uses the same amount of current as head-only electrical stunning but is operated at higher frequencies (1,000 to 2,000 Hz) with a square waveform rather than sinusoidal. It can be applied over the full body instead of only the head and because of high frequencies, it does not cause heart failure or death of the animal, convulsions associated with muscle activity, and consequent electrical immobilization for operator safety can be avoided (Simmons et al., 2006; Fuseini et al., 2017). From an animal welfare point of view, this technique is most preferred as head-to-body is reliable and causes no pain to the animal (Anil et al., 2006; Fuseini et al., 2018).

#### Negative electrical stunning outcomes.

The use of electric stunning for humane treatment of animal has received criticisms from Muslim opponents of stunning as they argue that stunning may result in the death of some animals. This concern can be minimized by using electrical head-only stunning commonly used for sheep and cattle, by maintaining the current, and checking each animal after stunning if it has been done right or not. This can be evaluated by checking if animal is still breathing, any vocalization, and remaining eye reflexes. However, electric water bath stunning commonly used for poultry is a concern from the animal welfare, meat quality, and religious compliance points of view (Rao et al., 2013).

The shackling and inversion of the birds, prestun shocks before stunning, and inappropriate current monitoring while passing through the head, body, and legs through stainless steel shackles are some of the issues with this technology (Lines et al., 2012). From a Halal perspective, the animal must be alive at the time of slaughtering, after the water-bath electric stunning. Some birds with low resistance may receive more current than required and are more likely to die prior to exsanguination and hence it is questionable if this is compatible with Halal meat production requirements (Fuseini et al., 2018).

### Mechanical (Percussive) Stunning

The fundamental principle of mechanical stunning includes the transfer of kinetic energy to the brain through a moving object which brings about the dysfunction or destruction of neurons followed by subsequent unconsciousness (Finnie et al., 2002). Mechanical stunning of the animal is accomplished by utilizing a penetrating captive bolt or a nonpenetrative percussion stunner (Vimini et al., 1983).

Mechanical stunning is defined as a stunning technique that uses an apparatus to cause a concussion within the animal’s forehead. Some previous literature included gunshot or free bullet and high-pressure water jets within the scope of penetrating stunning techniques. The high-pressure water jets method is experimental. With the penetrative captive bolt air pressure or a blank gun-powder cartridge drives a steel bolt into the middle of the animal’s forehead. Nonpenetrative captive bolt stunning is done the same way except that the end of the bolt has a wide mushroom-shaped head that cracks the skull rather than penetrates it. The shape of the 2 different types of bolts determines their properties (Blackmore, 1979; Daly et al., 1985, 1986).

The nonpenetrating captive bolt stunning procedure usually starts by moving the animal into a restraint box after which the head of the animal is restrained and the operator fires the bolt correctly to the previously identified position on the forehead of the animal which will cause unconsciousness of the animal immediately (Finnie et al., 2002; Grandin and Voogd, 2020).

The efficiency of mechanical stunning is not well documented regarding animal welfare and quality of meat, although it is generally expected to have harmful effects on the animal’s well-being as it can cause damage to the skull if not done properly. All of these electrical and mechanical techniques may have some human safety concerns, and, therefore, necessitate training, practice and the development of expertise. It should not be done by inexperienced individuals (Daly et al., 1986). Mechanical stunning methods are delivered effectively if the target area and the direction of the percussive bloware positioned correctly and sufficient kinetic energy is transferred. This requires proper dry storageof any gun powder cartridge or maintenance of the air–pressure system along with the device itself (Daly et al., 1985).

Many studies confirmed that penetrating captive bolt stunning leads to instant unconsciousness. Lambooy and Spanjaard (1981) using electroencephalogram (EEG) measurements concluded that the frontal penetrative captive bolt stunning almost definitely ensured instant unconsciousness because of the EEG wave generated directly after stunning. Another study, usingsomatosensory and visual evoked responses, determined that penetrating captive bolt stunning in sheep produced an instant and reflective brain damage that causes immediate insensibility of the animal. Gregory and Shaw (2006) did an abattoir survey and concluded that it is possible to obtain almost 100% successful penetrative captive bolt stunning with a single shot. Moreover, the small frequency of recovery means they felt that this method could be considered as a part of a humane slaughter process for cattle.

**Fig. 1:**
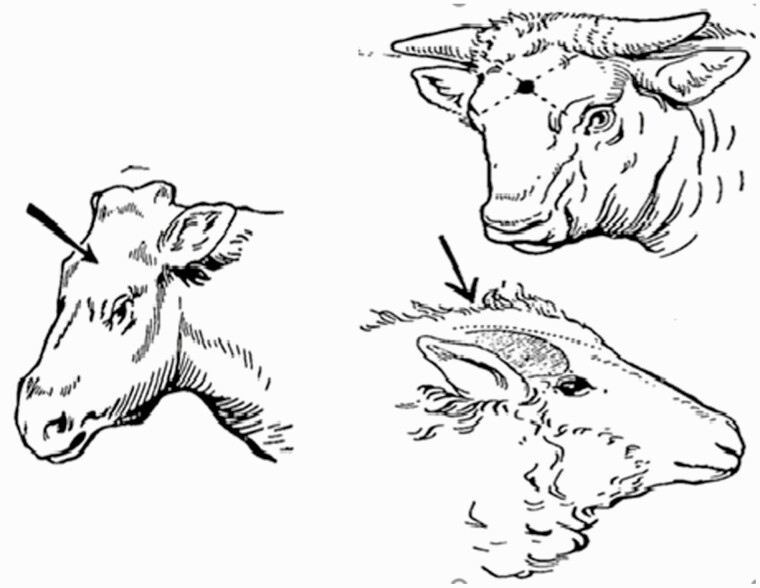
From the Schermer mechanical stunning apparatus instruction booklet, Alpha International Corp., New York, NY.

In a comparison study of penetrative captive bolt stunning with nonpenetrative captive bolt stunning by Grandin (2020b), it was determined that plants having an effective systematic approach toward good captive bolt stunning practices usually obtain 96% to 98% successful results and animals are rendered unconscious with a single blow causing irreversible damage to their brain (Grandin, 2020a). The correct placement of the stunner, sufficient bolt velocity, and accurate air supply of captive bolts to maintain pressure specified by the manufacturer is crucial for the success of percussive stunning on the first shot because if the animal fails to go down from the initial attempt it will become more problematic to stun the second time. Some plants routinely shoot animals twice to ensure the successful application (Grandin, 2020b). Figure 1 shows the correct frontal position for different types of animals. (The same position is used for nonpenetrating captive bolt stunning but requires that the accuracy of placement is greater, which is why the use of a head holder is strongly recommended, i.e., to keep the animal in position as the stunner is aligned.)

The effectiveness of any mechanical stunning device is dependent on the selection of the proper anatomical site and directional aim of the device. Coore et al. (2004) in a study found that 4% of the venous blood sample of cattle (95% confidence interval, 1.6% to 9.8%) stunned using a penetrative captive bolt stunner and from 2% (95% confidence interval, 0.6% to 7%) of cattle stunned using a nonpenetrating captive bolt stunner showed brain tissue fragments or raised levels of a maker protein for brain tissue in the blood. Lambooij et al. (1999) concluded experimentally that a modified captive bolt stunning method using air pressure had benefits over electrical water bath stunning for broilers. Also, the type of stunning and restraining treatment significantly affected the meat quality. Table 1 summarizes the suitable stunning methods for different types of animals such as livestock and poultry.

**Table 1:** Recommended mechanical stunning method(s) for different categories of animals (Nakyinsige et al., 2013)

Type of animals	Recommended Halal stunning methods
Cattle, steers, heifers	Nonpenetrating captive bolt, head-only electrical stunning
Sheep	Nonpenetrating captive bolt or electrical head-only stunning
Goats	Head-only electrical stunning
Turkeys, chickens, Halal birds	Water bath electrical stunning, gas stunning
Broilers	Nonpenetrating captive bolt or electrical head-only stunning
Ostrich	Head-only electrical stunning
Rabbits	Nonpenetrating captive bolt or electrical head-only stunning

#### Nonpenetrating captive bolt stunning.

The nonpenetrating captive bolt stunner is used almost exclusively on cattle and especially for Halal slaughter in most of western and European countries (Anil et al., 2004). Different types of nonpenetrating stunner are used, among which the mushroom head is the most common one. 

The “mushroom” is a convex metal disk ~4 cm in diameter and the impact is applied to the forehead (Gregory, 2007; EFSA, 2004). Newer models of the Jarvis pneumatic mushroom head nonpenetrating stunner are designed for faster bolt speed with larger stunning rod diameter to provide more accurate head contact and less skull damage. Trials with the Jarvis nonpenetrating stunner found it to be more effective on Zebu type cattle with very short hair.

The plant where the experiments took place had a head holding device to hold the head of the animal. The stunner only fractured the skull without breaking the skin and temporary unconscious occurred (Oliveira et al., 2018). The factors affecting the delivery of an effective nonpenetrative captive bolt are: (1) stun operator’s skill level (experience and training), the operator’s attitude toward the task and to animals and the operator’s focus (attention and fatigue); (2) facilities and equipment (movement up to and into the restraint), flooring (slipperiness), and distractions (lighting, noise, etc.); and (3) animal behavior, temperament, and anatomy (hair/wool, skull shape and thickness, scalp, neutral tissue).

It has been observed that the effective application of nonpenetrating captive bolt (mushroom-shaped) stunning powered by a cartridge and delivery of a sufficient blow to the frontal/parietal bones, causes acceleration and deceleration of the head and creates shear and rotational forces in the brain that leads to the immediate unconsciousness due to a brain concussion (EFSA AHAW et al., 2020). Observations showed that the nonpenetrating stunning was less efficient on wooly head cattle such as Herefords compared with short haired cattle. Changes are reversible and the animal can become conscious again after some time (Oliveira et al., 2018). This is an unreliable technique for mature cattle, calves, and sheep, and should only be used for Zebu-type cattle (Oliveira et al., 2018; Collins et al., 2020).

#### Gunshot or free bullet.

Gunshot or free bullet stunning could be considered as a different form of penetrative captive bolt stunning without restraint before stunning. Historically, the gunshot or free bullet was used for euthanasia of animals on the farm or in the wild. To kill the animal immediately without pain, an appropriate pistol and gunshot with adequate speed, power, and size needs to be selected to penetrate the brain and cause skull destruction. Different types of bullet are used for different animals. For example, for young animals a long rifle 22 caliber solid point bullet is appropriate while this bullet may not penetrate the brain in mature animals. The proper distance between the firearm and the head of the animal may be 56 to 88 cm (2 to 3 ft) for best results (Anil et al., 1995; Nakyinsige et al., 2013).

In most euthanasia cases using gunshot, the target animals are incapacitated or “down” so the chances for accurate positioning of the bullet are easier. Alternatively, for a potentially dangerous animal or one in motion, it may be necessary to shoot from a distance, which has its limitations, and the ideal target sites are the head, neck, or lower thorax (Roth et al., 2007).

Special equipment is required to determine whether an animal is actually unconscious and unable to feel pain. There are 2 physiological measurement methods known and used; the EEG, which records the brain’s electric pulses, and the electrocardiogram, which records the heart’s electric pulses. The ECG should not be considered as a sole indicator of unconscious, because an animal could be conscious for several seconds after the heart pulses have stopped and an animal with a heart pulse may be unconscious (Schulze et al., 1978).

#### Mechanical stunning and Halal requirements.

An animal needs to be alive at the time of Halal slaughter. Captive bolt (penetrative) stunning is unacceptable from a Halal perspective as an animal will not recover fully if slaughtering is not doneafter such stunning. Gregory (2007) found that the concussion caused by the captive pins from the captive bolt is an efficient method that alters brain function instantly to stun the animal and these changes can be permanent as confirmed by studying the evoked potentials. Evoked potentials involve the electrical potential in the brain that takes place as a result of an external stimulus. Correctly stunned animals with the penetrating captive bolt stunning instantly lost their evoked potentials and caused irreversible impacts. The nonpenetrating stunner, although it does not penetrate the brain, has similar EGG patterns as that of the penetrating captive bolt stunning that is an indicator of reversible unconsciousness and animal may revive unless it is bled promptly. Therefore, its use has been discouraged for cattle slaughtering because of the uncertainties of its efficacy and welfare concerns for the animal (Farouk et al., 2014). The European Council Regulation (EC No. 1099/2009) that entered into force in January 2013 forbid the use of nonpenetrating captive bolt stunning except for animals that are <10 kg body weight. In a comparative study of penetrating and nonpenetrating captive bolt, and electrical stunning, pneumatically- or cartridge-operated penetrating captive bolt stunning was found to elevate the risk of hematogenous spread of tissue from the central nervous system. The spread of central nervous system tissue represents a danger to the general well-being of humans as it might permit diseases such as preclinical bovine spongiform encephalopathy (BSE) to be found elsewhere in the animal (e.g., in the meat) (Anil et al., 2002). Specifically, stunning with the captive bolt can cause brain material to pass into the lungs through the jugular veins (Gregory, 2005).

There has been renewed interest in the use of nonpenetrating captive bolt due to the risk of spread of BSE when using captive bolt stunning. The removal of the bolt injected into the brain minimizes the spread of brain or spinal cord tissues that could possibly contaminate other parts of the body and brain tissues in contact with the penetrating bolt can contaminate slaughterhouse equipment (Grandin, 2020a). Captive bolt stunning has been modified to limit the transfer of BSE prions to the blood stream, although uncertainty that this has been properly addressed remains (Pitardi et al., 2013; Grandin and Voogd, 2020). Nonpenetrating captive bolt stunning for cattle has been accepted by some Islamic organizations. Many Halal controlling bodies (HCB) have expressed their concern about using irreversible stunning methods because of the fear of the animal’s death prior to slaughter.

While effective use of nonpenetrating captive bolt stunning requires much more accurate aim as it requires head holding equipment, it is more likely to reduce the spread of brain material as fracturing is minimized. A mushroom head with larger diameter might be more effective with less fracturing than a mushroom head of smaller diameter (Grandin, 2020a).

Some Muslims in Sweden use captive bolt stunning or electrical stunning that results in death of the animal because of the requirements of Swedish law that prohibits the use of traditional Halal slaughter methods (Fuseini et al., 2016).

Many Muslim religious authorities do not want stunning to kill the animal. The heart continues to beat for several minutes after both types of captive bolt stunning. If the animal is required to bled quickly following the stunning, the heart should be beating at the time of bleeding. Beating of heart helps to drain the blood out of animal’s body quickly and completely. If bleeding is delayed, the heart will stop working due to the lack of oxygen and blood will not be drained out of the animal body which is a requirement of Halal slaughter. Properly done captive bolt stunning causes the heart to stop beating immediately (Grandin and Voogd, 2020). Irreversible effects of penetrative captive bolt stunning on the brain can be a contributing factor toward the animal’s death and may not be dependent on the throat cut and exsanguination to cause death as required for the Halal slaughter of animals (Zulkifli et al., 2014).

There has been a lot of doubt and concern regarding the use of any form of mechanical stunning for Halal meat production, but the doubt is stronger for penetrating captive bolt stunning. As Islam suggests avoiding the doubtful things, the authors suggest avoiding captive bolt mechanical stunning for Halal meat slaughter.

#### Positive effects of using different mechanical stunning methods before slaughter.

The effective stunning method should not make the animal feel any pain and distress. The shackling and hoisting of a sensible animal or prolonged sensibility during exsanguination (bleeding) should be avoided. The efficiency of the stunning method is determined by the number of successful stuns with the first shot, i.e., >95% stunned with the first shot is a minimal requirement and is part of the North American Meat Institute standard for religious slaughter as originally developed by Dr. Temple Grandin. This standard is generally the de facto standard globally. Captive bolt stunning is usually safe for operators and bystanders. Also, after the initial investment, continued operation is inexpensive. Whereas, to use gunshot properly, 1 bullet is needed each time and will cause immense brain damage along with instant unconsciousness. Captive bolt stunning is also economical. Properly done captive bolt stunning has been shown to provide 95% to 98% accurate results (Grandin, 2020b). It does not involve close contact with the target animal. In a comparison study, 3 methods of preslaughter stunning, i.e., no stunning, head-only electrical stunning, and percussive captive bolt stunning were assessed for Halal meat quality. Results showed that percussive stunning improved meat quality attributes such as pH, water holding capacity, texture, color, and consumer sensory attributes when compared with the other 2 methods, but it cannot be applied for Halal animals (Önenç et al., 2004). Nonpenetrating captive bolt stunning, which is accepted by many Halal religious authorities, eliminate the risk of brain cell spreading that can cause BSE and possibly other diseases to be transmitted to the meat and equipment contamination. When done properly, it is also reversible, as the animal can be revived. Accurately done nonpenetrative captive bolt stunning, however, requires more work but once done right fulfills the criteria according to many authorities for Halal stunning (Oliveira et al., 2018).

#### Negative effects of using different mechanical stunning methods before slaughter.

When captive bolt is done for Halal slaughter, many Muslims religious authorities do not support captive bolt stunning that can kill the animal. An animal needs to be alive at the time of slaughter. In addition, the operator needs to be near the animal, and it should be confined. Furthermore, the procedure should not be started when the animal is moving its head.

One disadvantage of using gunshot is that it may be dangerous because of the potential ricochet of the bullet. Therefore, the operators need to make sure that they take extra care in positioning themselves and others during the procedure. Another disadvantage is, when dealing with agitated animals, the operator may not be close enough to precisely hit the target animal. It can also cause intracranial hemorrhage, cracks in the skull and brain tissue release (Farouk, 2013).

Nonpenetrating captive bolt stunning requires different adjustments according to the species of animal. Observations have shown that the nonpenetrating stunner is less effective on cattle with more hairs than with a less haired one. Its use is also discouraged for adult cattle and can be used only for certain types of animals (Collins et al., 2020).

### Gas Stunning

Carbon dioxide was first proposed in the mid-1950s for the preslaughter stunning of poultry (Raj et al., 1996). Gas stunning, also called controlled atmosphere stunning, has become more common during the last 20 years because of its advantages in animal welfare (although some argue that there is an aversive stage that suggests the animal is being agitated) and product quality compared with the water-bath method. Carbon dioxide is a colorless gas and it is heavier than air. Therefore, stunning needs to be done in a pit or in a closed apparatus (Raj et al., 1994).

The inhalation of high concentrations of carbon dioxide induces hypercapnic hypoxia. Therefore, changes in the blood occur including lowering the pH, increased carbon dioxide partial pressure, decreased oxygen partial pressure, lack of oxygen saturation, and lower bicarbonate concentration. As a consequence, the pH of the cerebrospinal fluid will decrease resulting in the animal losing consciousness. Gas stunning with high concentrations of CO_2_ (70% to 95% CO_2_) is mainly used for turkeys, chickens, and rabbits. Animals are stunned by exposing them to a concentration of CO_2_ (70% to 98%) and/or a mixture of N_2_ (80%) and CO_2_ (20%) (Llonch et al., 2012; Dalmau et al., 2016).

Rabbits lose their consciousness on average in 30 s when exposed to at least 80% CO_2_ but they should be exposed to gas concentration for at least 2 min to ensure enough duration of unconsciousness until slaughtering. As gas stunning is done in larger batches, the interval between the end of exposure to the gas and sticking of the last animal might get longer when compared with other stunning methods so requires longer exposure of the animal to gas when compared with electrical stunning (EFSA AHAW et al., 2020). Sometimes animals can be killed using higher concentrations of carbon dioxide (Raj et al., 1994). Sometimes other gases are used such as argon, but other gases are generally more expensive than CO_2_ and are not normally used by those doing gas stunning of animals.

#### Gas stunning from the Halal perspective.

Modified atmosphere stunning (MAS) is another form of gas stunning where livestock are exposed to a specific amount of carbon dioxide through tunnels, containers, pits, or sealed buildings. This happens sometimes in 2 phases: (1) livestock are exposed to a mixture of gases having at least 40% carbon dioxide by volume in air and (2) in a second phase after the animal became unconscious, higher concentration of carbon dioxide is sprayed into the system (EFSA AHAW, 2013). Another approach is using a mixture of gases (including the inert gases: argon, nitrogen, or helium) with carbon dioxide to promote anoxia (ideally without any aversion), or sometimes only inert gases are used. Low atmospheric pressure stunning is another type of modified atmosphere system that is done by lowering the atmospheric oxygen and promoting hypoxia such that the animal loses their sensibility because of brain function inhibition (EFSA AHAW, 2013).

Carbon dioxide stunning does not guarantee the absence of discomfort and pain in animals. However, the absence of gas as with most animals stunning methods has both advantages and disadvantages from the animal welfare perspective (Joseph et al., 2013).

Not enough work has been done on the use of gas stunning for Halal meat production. A full assessment of the potential physical and brain impact of gas stunning as an alternative to electrical and mechanical stunning methods is needed to determine whether it is an improved stunning method and is consistent with Halal requirements and meat quality. Animal must be alive after gas stunning and changes need to be reversible as a requirement of its use for Halal slaughtering. A major advantage is the ability to do group stunning, i.e., to leave poultry in their transport cages until after stunning, which becomes a major improvement in eliminating pre-handling stress (Raj, 2006).

#### Positive effects of using different gas stunning methods before slaughter.

Stunning using CO_2_ does not require prior restraint for animals going into a gondola where the gas will be added (Ismatullah et al., 2015). Because this method requires less handling of the animals, it may allow more animals to be stunned and slaughtered per hour than with an electrical stunning method. Gas stunning strongly decreases the problem of broken bones, and it decreases hemorrhaging of the leg and breast muscles. Also, it increases the decline of pH postmortem, so the quality of the carcass is improved compared with electrical stunning (Turcsán et al., 2003). Such meat is considered to be of better quality, and there is an absence of convulsions after stunning. It is less sensitive to differences in bird size and conformation compared with water-bath stunning while avoiding shackling birds prior to electrical stunning (Grandin, 1980; Grandin, 2010; Berg et al., 2015). Therefore, gas stunning is considered a more humane technique of immobilizing poultry than electrical stunning. Gas stunning is preferred because it results in fewer blood spots in the meat and fewer hemorrhages on the surface of the carcass (Turcsán et al., 2003; Nowak et al., 2007).

#### Negative effects of using different gas stunning methods before slaughter.

Several studies have shown that CO_2_ is highly aversive to chicken and turkeys and can be a cause of significant pain during stunning when exposing them to high concentrations of the gas. This method is more stressful for the animals when compared with electrical and captive bolt stunning when they are done correctly (Llonch et al., 2012). Gasping is an indicator of respiratory distress that is associated with CO_2_ stunning which is perceived as an alarming response but is also related to unpleasant reactions to gas inhalation (EFSA AHAW et al., 2020). LIonch et al. (2012) reported a higher percentage of rabbits gasping when exposed to 90% CO_2_ than to 80% N_2_ and 20% CO_2_.

The major disadvantage of gas stunning is that it does not make the animal unconscious immediately; instead, unconsciousness occurs progressively (Berg et al., 2015). Also, birds stunned using gas cannot recover consciousness and are killed by suffocation, which does not meet Halal and kosher requirements. It is known that high concentrations of CO_2_ (>80%) for a long duration (more than 45 s) are damaging to the respiratory tract, so this might not be an appropriate method for the animal’s welfare. High concentrations (and duration) of CO_2_ might also make the animal stiff, which will decrease the bleed-out, which is problematic for Halal meat (Llonch et al., 2012). However, carbon dioxide’s effectiveness depends on concentrations and exposure times, which may not result in efficient stunning, and animals might recover from the stunning before they are cut for bleeding.

There is also a lack of information concerning why there is variability among individual animals in their behavioral reactions to CO_2_ gas, and the impacts of handling and stress before gas stunning. Finally, the existing industrial gas stunners are large and may have high investment costs (Ismatullah et al., 2015).

## DEBATE ABOUT SLAUGHTERING WITH AND WITHOUT STUNNING

Many countries and organizations permit the religious slaughter of animals without stunning. However, it remains extremely controversial from the animal welfare point of view (Grandin, 2010). The animal welfare problems during slaughter without stunning include the stress of the restraint, the feeling of pain while slaughtering, or excessive distress during bleeding such as blood aspiration into the lungs (Gregory, 2005).

Grandin and Regenstein (1994) on some studies of observed more than 3,000 veal calves and cattle in 3 different slaughter plants in the United States that were using the upright restraint system where animal feel little or no response while their being throat cut. A slight shudder was noticed when the blade first touched the throat, but this was significantly less vigorous when compared with response of an animal when an ear tag was put on. No further response was observed as the cut proceeded. It seems like the animals were not aware that their throat was being cut.

According to Grandin and Regenstein (1994), if the details mentioned about the kosher slaughter knife design (twice the length as the width of the neck and the absence of any nicks), then cutting of the neck when done properly seemed to prevent animal from reacting to the shechita cut. However, slaughtermen using an inappropriately less than sharp knife results in compromising of animal welfare that is also reflected in lower meat quality (Grandin and Regenstein, 1994). The brain function of adult cattle after preslaughter captive bolt stunning was compared with cattle slaughtered without stunning visually and with somatosensory evoked potentials. It was observed that the nonstunned cattle resulted in greater variability with respect to the loss of evoked reactions (20 to 126 s) compared with the animals that had been prestunned (Daly et al., 1988). However, these studies were done using a restraining pen that has since been rejected as unacceptable.

Various studies by Johnson et al. (2012) summed up the effect of slaughtering without stunning and found out that slaughtering without stunning is associated with pain from the point of slaughter until the unconsciousness of the animal which also can lead to blood aspiration into the lungs while the animal is still conscious. After slaughtering, breathing continues during early bleeding of the nonstunned animal while a stunned animal shows no signs of breathing. Breathing during initial bleeding as the animals were not fully unconscious can cause airway irritation because of the presence of the blood in their lungs (Gregory et al., 2009). Unfortunately, this study was not done with an appropriate knife as discussed above along with other limitations from the religious point of view so its relevance to kosher and well-done Halal practices are questionable.

Other concern about the nonstunned animals is the pain they experience depending upon the rate of loss of consciousness following the slaughter. According to different studies, false aneurisms were found in the carotid arteries at the cardiac and cephalic ends that were the cause of prolonged (≥60 s) consciousness of slaughtered animal. However, when done correctly, kosher and Halal slaughtered cattle will take an average of 33 s to become unconscious and an animal that is not unconscious at 40 to 60 s ought to be stunned. False aneurisms cause sustained consciousness and can form as early as between 7 and 21 s following Halal animal slaughtering, hence causing improper bleeding out of the animal (Gregory et al., 2010; Gregory et al., 2012).

On the other hand, head-only electrical stunning is widely accepted from an animal welfare viewpoint. In a review article, electrical stunning was compared with unmodified human ECT instead of epilepsy and found that electrically stunned animal also showed some negative impacts of ECT, e.g., muscular pain, serious anxiety, and high frequency of fractures. Inappropriately or appropriately stunned animals experience “sub-convulsive stimulation,” events, which may possibility occur even under the best circumstances. Such an event would compromise the welfare consequences when compared with nonstunned animals (Zivotofsky and Strous, 2012).

According to some scholars, electrical stunning inhibits proper blood loss that is a requirement of Halal meat, as no blood should be present in Halal slaughtered meat. Studies showed that total blood loss without stunning is not different from blood loss using pre- or poststunning methods (Anil et al., 2004; Gomes et al., 2009). In a study by Khalid et al. (2015), the blood loss of lambs during Halal slaughtering without stunning, electric head-only stunning and post-cut electric head-only stunning were compared and they found a small difference in blood loss after 10 and 60 s which was lowest in slaughtering without stunning compared with the other 2 types of slaughter but there was no significant difference among the 3 treatment after an interval of 90 s.

According to Addeen et al. (2014), all forms of stunning have a negative impact on the quality of meat, and animal do not feel pain when slaughtering is done without stunning as blood can be drained completely out of the animal’s body because it is considered an accuracy standard for good quality of meat. It can be interpreted that the animal slaughtered without stunning produces a better quality of meat for the human body if they are consumed. In the Halal meat cutting process, it is believed that producing full bleeding is quite perfect, and that it can be beneficial in extending the shelf life and guaranteeing of meat quality.

## CONCLUSIONS

Halal meat consumption is considered as a form of worship for the followers of Islam as a commandment from Allah (God). The importance of the requirements for Halal slaughtering of animals is clear from the Quran and the Hadiths (sayings and actions of the Prophet (peace be upon him)), the most important of which is that an animal must be alive during the time of slaughter. This creates a controversy and debate in various Muslim communities about the acceptance of preslaughter stunning as Halal, which is mainly because of the fear of the animal’s death during stunning, prior to the actual Halal slaughter. To solve this issue, responsible Halal certifying bodies that accept stunning have rules specifying the requirements of stunning, i.e., simple and reversible stunning are generally accepted only when there is a procedure to check/detect dead animals. However, there always have been questions about the accuracy of the system used to detect animal death in a slaughterhouse because sometimes animal develop false aneurysms that can create misleading results. Irreversible stunning methods can cause fibrillation of the heart or significant brain damage as in mechanical stunning and water-bath stunning of poultry continues to be of concern because of the difficulty of assuring the absence of animal death, especially in cases when there is a time-lapse between stunning and slaughtering.

To avoid a stressful killing method, there is a need to improve animal welfare strategies on a practical level that involves the proper training of slaughtermen and other workers in the slaughter area, having well-maintained equipment, a better attitude about the treatment of animals, and good working conditions so that workers want to do things right with strong support from management. Inappropriate current flow or improper electrode placement during electrical stunning, inadequate air pressure or wrong cartridge size in pneumatically operated percussion system, inaccurate shooting positions with captive bolt stunning and delayed gas induction to produce the anesthetic effect during stunning are all factors causing concerns related to animal welfare. These issues can be addressed if everyone, including management, are committed to doing things right.
